# An Excel program for calculating statistics presented in Marfak et al.’s article ‘Improved RIDIT statistic approach provides more intuitive and informative interpretation of EQ-5D Data’

**DOI:** 10.1186/s12955-021-01745-5

**Published:** 2021-04-05

**Authors:** Abdelghafour Marfak, Ibtissam Youlyouz-Marfak

**Affiliations:** 1 National School of Public Health, Rue Lamfadel Cherkaoui, Madinat Al Irfane, BP-6329 Rabat, Morocco; 2 Laboratory of Health Sciences and Technology, Higher Institute of Health Sciences, Hassan First University of Settat, Settat, Morocco

**Keywords:** Improved RIDIT, EQ-5D, Quality of life, Absolute Risk Reduction, Absolute Risk Increase, Odds ordinal

## Abstract

The objective is to present and share an Excel program that we have developed to perform statistical analyses based on the Improved RIDIT approach of Marfak et al.’s article ‘Improved RIDIT statistic approach provides more intuitive and informative interpretation of EQ-5D Data’.

**Supplementary Information:**

The online version contains supplementary material available at 10.1186/s12955-021-01745-5.

## Introduction

In our article ‘Improved RIDIT statistic approach provides more intuitive and informative interpretation of EQ-5D data’ [1], we presented the theoretical bases of the Improved RIDIT method as well as its application for studying the effect of a disease or a treatment on the quality of life evaluated by the EQ-5D instrument.

Herein, we present and share with the scientific community in the field of health and quality of life an Excel program consisting of two separate files for performing the Improved RIDIT calculation and interpretation for both EQ-5D-3L (file: ‘ImprovedRIDIT-Calculation-EQ-5D-3L.xlsx’) and EQ-5D-5L (file: ‘ImprovedRIDIT-Calculation-EQ-5D-5L.xlsx’) versions. Each of the two Excel files comprises 7 sheets. The first sheet (Explanation & Interpretation) shows how to use the Improved RIDIT Excel program and how to interpret the results. Five sheets named MO, SC, UA, PD and AD for calculating the Improved RIDIT statistics for mobility, self-care, usual activities, pain/discomfort and anxiety/depression dimensions, respectively. The sheet (Summary Statistics) presents graphical and numerical distributions of responses obtained from the two EQ-5D samples. Also, this sheet summarizes the results of the five dimensions in a table, which can be used for results publishing. The *p*-values are corrected for multiple testing by the Bonferroni-Holm procedure [2]. The following steps show how to use the Improved RIDIT Excel program:

1. First, if data come from the EQ-5D-3L version, use the Additional file 1: ‘ImprovedRIDIT-Calculation-EQ-5D-3L.xlsx’ file; else use Additional file 2: ‘ImprovedRIDIT-Calculation-EQ-5D-5L.xlsx’ file if data come from the EQ-5D-5L version.
2. In each MO, SC, UA, PD and AD sheet in the Excel file, remove all data in the columns ‘Expected to be in better health’ and ‘Expected to be in worse health’ used for demonstration.
3. In the column ‘Expected to be in better health’ enter your own data from the sample that you expect individuals to be in better health status.
4. In the column ‘Expected to be in worse health’ enter your own data from the sample that you expect individuals to be in worse health status.
5. Once data entry is complete, the results are displayed on the same sheet under using (MO, SC, UA, PD and AD).
6. Do steps 1–3 for the five sheets (MO, SC, UA, PD and AD).
7. Finally, to use the ‘Summary Statistics’ sheet, copy results and past in a word file.

## Supplementary Information

**Additional file1:** Excel program for calculating Improved RIDIT statistics for EQ-5D-3L data.

**Additional file2:** Excel program for calculating Improved RIDIT statistics for EQ-5D-5L data.

## Data Availability

The Excel program presented in this letter is available in supplementary materials as two separate files: ‘ImprovedRIDIT-Calculation-EQ-5D-3L.xlsx’ file (for the EQ-5D-3L version) and ‘ImprovedRIDIT-Calculation-EQ-5D-5L.xlsx’ file (for the EQ-5D-5L version).

## Abbreviations

ARR: Absolute Risk Reduction
ARI: Absolute Risk Increase
